# Between-session reliability and sensitivity of force production characteristics across various force-time points during the isometric single-leg long-lever bridge

**DOI:** 10.1371/journal.pone.0350197

**Published:** 2026-05-22

**Authors:** Adam E. Sundh, Nicholas J. Ripley, A.J. Lamb, Conor J. Cantwell, Paul Comfort

**Affiliations:** 1 School of Health and Society, University of Salford, Salford, United Kingdom; 2 Chicago Bears Football Club, Lake Forest, Illinois, United States of America; 3 Department of Athletics, William & Mary, Williamsburg, Virginia, United States of America; 4 School of Medical and Health Sciences, Edith Cowan University, Joondalup, Australia; Universidade de Aveiro Escola Superior de Saude de Aveiro, PORTUGAL

## Abstract

The purpose of this study was to assess the between-session reliability of peak force and early force-time variables in a unilateral isometric hamstring assessment (isometric long-lever bridge). The sample consisted of 30 collegiate male athletes (age: 19.4 ± 1.3 years; height: 1.79 ± 0.1 m; body mass: 80.4 ± 10.3 kg) with no history of recent hamstring injury (<6 months) who volunteered to participate in the study. All participants performed 3 unilateral maximal voluntary isometric contractions (MVIC) with their heels on force plates and shoulders elevated 15.24 cm, while their hips were secured with a loaded barbell. Data was analyzed to assess the reliability of force values at 50, 100, 150, 200, 250 ms, and peak force (N) across three repeated sessions. Moderate-good relative and absolute reliabilities were observed across all early force-time variables with lower bound ICC values ≥ 0.51 (0.51–0.71) and point estimate CV% ≤ 12.65% (7.63–12.65), with moderate relative (ICC ≥ 0.74; range 0.74–0.92) and absolute (CV% ≤ 6.50%; range 6.48–6.50) reliability for peak force. Trivial to small effect sizes (*g* ≤ 0.4) were observed between sessions. These findings suggest that the isometric single-leg long-lever bridge (SL-LLB) can be used to reliably monitor peak and rapid force generating capacities to track performance changes over time due to training adaptations as well as acute or chronic fatigue.

## Introduction

Valid and reliable measurements of force production characteristics are of critical importance when measuring strength outcomes and guiding rehabilitation strategies in sport [1], especially considering researchers in the field of the sports sciences may primarily want to assess how various interventions or treatments may alter the outcome of the assessment [2]. Detecting small changes may provide pivotal information within performance and rehabilitation protocols [3], therefore ensuring the reliability of any assessment is of paramount importance. A non-reliable test may not be suited to accurately measure long-term outcomes as they may display measurement error rather than true changes in performance [1]. Thus, any assessment that a sports practitioner wants to undertake must first be established as a reliable assessment, with a low measurement error, to ensure proper conclusions are being drawn based on the results, otherwise faulty assumptions may lead to uninformed decisions and poor outcomes [4].

In recent years, more and more strength assessments have been conducted in applied sports performance settings to evaluate performance [5,6] and potential injury risk [7], with one such area of interest being the evaluation of hamstring strength [8–12]. This interest appears to have grown recently considering the hamstring musculature, especially the long head of the biceps femoris (BF_LH_) has been shown to be the most commonly injured muscle across a variety of sports [13–16]. Perhaps more importantly, hamstring strain injuries (HSI) appear to be increasing across competitive sport [17], and make up a large portion of the overall injuries in football code sports [18–22]. Hamstring strength has been suggested to be a key indicator for mitigating HSI’s [7,23] supporting the broader theory that muscular strength is a critical component in injury mitigation [24]. This suggests that injuries appear to occur due to an insufficient capacity for voluntary force production relative to the required force application [25]. Thus, the assessment of force production characteristics, especially at areas prone to injury, could be valuable when monitoring for identifying higher risk individuals or assessing the efficacy of a program, and could further be applied to ensure appropriate return-to-play protocols are being followed [26]. However, a single measurement may not provide the full picture, emphasizing the need for continuous monitoring to track changes longitudinally over the course of a season or training program [27]. This may create challenges given that small changes in testing parameters may drastically impact results and can therefore result in incorrect beliefs if not meticulously standardized [4].

Considering the most commonly injured hamstring muscle (BF_LH_) is biarticulate [28], the assessment of simultaneous knee flexion and hip extension may be warranted. One such isometric hamstring assessment, termed the 90:20 (referring to a standing assessment consisting of 90° of hip flexion and 20° of knee flexion) has already been researched [29–32], but appears to demonstrate less reliability than other proposed isometric hamstring assessments [12], and is performed with 20° of knee flexion. Considering the BF_LH_ may be maximally active at 30° of knee flexion [33], and additional hip flexion may place excessive strain on the hamstrings [34], the implementation of a long-lever alternative against a fixed object that emphasizes knee flexion and hip extension executed at a 30° degree knee angle may be warranted to emphasize maximum force production of the BF_LH_ [4]. Thus, the SL-LLB, may provide a valuable alternative to the 90:20 isometric hamstring testing protocol, consistent with a growing body of literature supporting its reliability and practical utility. While within-session reliability [4] and the consistency of asymmetry magnitude and direction have been examined [35], between-session reliability of early force-time variables specifically has yet to be established. Notably, Driggers et al. [36] demonstrated its application in professional baseball players in relation to HSI risk, further supporting the practical relevance of this assessment across competition levels.

Moreover, despite its relevance for performance, there is a paucity of research examining rapid force production characteristic in isometric hamstring testing [9]. This is particularly concerning given that reliability appears to decline during the earlier stages of force production [37], and that rapid force production characteristics seem to represent distinct constructs from late or peak force production [35], suggesting that assessing only peak force may overlook meaningful aspects of neuromuscular performance. Furthermore, the ability to rapidly produce force is critical to various actions like sprinting [38] and jumping [39] where initial force application plays a substantial role in performance and injury, this becomes particularly relevant in terms of hamstring function due to the high injury burden. Given the demand of the hamstrings to decelerate the tibia during the swing phase of sprinting [40] with knee extension angular velocities potentially exceeding 1200 deg/s [41], the capacity to rapidly produce force is essential. As such, understanding how the hamstring musculature performs during these early stages of force production could provide more relevant information for dynamic movements in sports and performance.

Therefore, the purpose of this study was to examine the reliability of force production characteristics across various force-time points in male collegiate athletics. It was hypothesized that the fixed-position of the isometric SL-LLB would yield good-excellent reliability, even at rapid force-time points compared to previously reported findings due to fixation of the hips leading to greater stability and therefore a greater ability to produce rapid force.

## Materials and methods

### Participants

A total of 34 participants competing in Division 3 collegiate athletics were initially recruited for the study, including football (American; n = 16), men’s soccer (n = 14), baseball (n = 3), and track and field (n = 1). However, due to scheduling conflicts, the baseball and track and field athletes (n = 4) were unable to complete all sessions. This resulted in a final sample of 30 athletes (age: 19.4 ± 1.3 years; height: 1.79 ± 0.1 m; body mass: 80.4 ± 10.3 kg). All participants were required to have had no sustained hamstring injuries within the preceding 6 months and suffered no catastrophic lower extremity injury in the 12 months preceding the study. Injuries were classified as any tissue damage that resulted in missed time from practice or competition, with catastrophic injuries defined as severe conditions causing an absence of more than six months. Organizational consent was obtained from a senior administrator within the athletics department prior to approaching any participants for the study. Participants were informed of the study’s objectives prior to its initiation and were required to provide written informed consent prior to participation. Ethical approval was granted by the University of Salford’s institutional review board (Reference no: 0902) in accordance with the declaration of Helsinki. An *a-priori* sample size calculation was performed using the sample size estimation method for reliability studies, developed by Borg et al [42]. The sample size was calculated based on peak force, assuming a lower bound ICC of 0.75 (minimal acceptable reliability), 0.90 (estimated reliability), 0.05 significance level, a statistical power of 0.80, and a three-repeated measures design, resulting in 24 participants required.

### Experimental design

A repeated measures cross-sectional design was used for comparative analysis of the reliability of the isometric hamstring assessment. Participants completed the testing procedures during their normal training hours on three occasions, separated by 48 hours each, over the course of 5 days, allowing for adequate recovery between sessions. Participants were recruited between 01/12/2024 and 20/01/2025, with all testing conducted within a single one-week period (27/01/2025-02/02/2025).

### Isometric single-leg long-lever bridge

**Fig 1:**
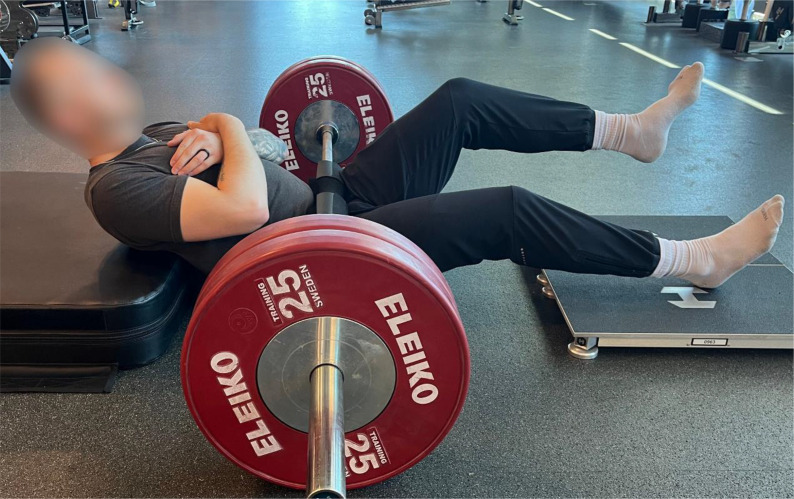
Representation of the Isometric Single-Leg Long-Lever Bridge Assessment.

The isometric SL-LLB assessment was performed using force plates (Hawkin Dynamics Inc., Portland, ME, USA) sampling at 1000 Hz, with data collected through the Hawkin Dynamics mobile software. While laying supine, the force plates were positioned on the ground, ensuring that the heels of the participants’ shoes were in direct contact with the plates while the inferior angle of the scapula rested on a 15.24 cm (6-inch) foam platform to elevate the torso while facilitating the intent of hip extension. To secure the lower body, a loaded barbell with a rigid foam pad was positioned at the inguinal fold, providing a stable point of resistance during the assessment. The test was conducted unilaterally with the hip positioned at approximately 135° ± 5° and a 30° knee angle maintained during each trial to maximize recruitment of the BF_LH_ of the assessed limb, while the contralateral limb was elevated off the ground and maintained a bent knee position (Fig 1). Each limb underwent three trials, during which the participants were cued to push as hard and fast as possible while driving their heels into the force plates and extend their hips into the loaded barbell for 3–5 seconds. Before initiating movement, participants were instructed to remain as still as possible to calculate limb weight and corresponding force-time data. If any trial within the same limb deviated by ≥ 50 Newtons (N) from the previous trial, it was discarded, and the trial was repeated until it was within the desired threshold. Trial repetition frequency was not formally tracked but did not interfere with protocol completion for any participant.

### Data analysis

Force-time data were initially collected at a sampling frequency of 1000 Hz and subsequently processed using Python’s SciPy package. To enhance reliability during rapid force production [43], a low-pass 4^th^ order Butterworth filter with a cutoff frequency of 50 Hz was applied to preserve integrity of the original signal, consistent with filtering parameters reported for this technology in previous research [44]. The onset of movement was identified as the point where force exceeds five standard deviations (SD) above the average force measured during the one-second weighing phase [45]. Force values at 50 ms, 100 ms, 150 ms, 200 ms, 250 ms, and peak force (N) were calculated from the onset of movement through each corresponding time interval. The mean force values for the three trials were calculated separately by left and right limb and used for subsequent analysis. Limb dominance was not recorded; given the heterogeneous sample spanning multiple sports, in which dominance patterns and their influence on force production may vary considerably.

### Statistical analyses

All statistical analyses were performed using the Python 3.12.7 programming language (Python Software Foundation, DE, USA). Data are presented as mean ± SD, with normality confirmed using the Shapiro-Wilk test. An alpha level of <0.05 was set *a priori*. Between session absolute reliability was determined using the coefficient of variation (CV%), calculated from the sample SD and 95% confidence intervals (CI). CV% was interpreted as follows based on the point estimate: < 5.00% (excellent), 5.00–9.99% (good), 10.00–14.99% (moderate), and > 15% (poor). Relative reliability was assessed using two-way, absolute agreement intraclass correlation coefficients (ICC; 3,1), with interpretations based on the lower bound of the CI: < 0.49 (poor), 0.50–0.74 (moderate), 0.75–0.89 (good) and > 0.9 (excellent) as suggested by Koo & Li [46]. The Standard Error of Measurement (SEM) which quantifies the precision of repeated measurements by estimating measurement error or variability in each test, was calculated using the following formula:


$$\begin{array}{c} SEM=SD_{pooled}\times \sqrt{\left(1-ICC\right)} \end{array}$$

The Minimal Detectable Difference (MDD) represents the smallest significant change that exceeds the error of the SEM, ensuring that observed differences are due to real change. The MDD was derived as:


$$\begin{array}{c} MDD=(1.96\times 2)\times SEM \end{array}$$

Where 1.96 corresponds to the critical value for a 95% CI and the √2 accounts for the error propagation between measurements, intended to predict an increase that should be reached in a longitudinal testing design to improve interpretability of results [47].

To assess the effect of the familiarization session on reliability, a repeated measures ANOVA (RM-ANOVA) with Bonferroni post hoc tests and Hedges’ g effect sizes were calculated. The magnitude of difference, as determined by Hedges’ g effect sizes, were interpreted following Hopkins’ [48] recommendations: ≤ 0.19 (trivial), 0.20–0.59 (small), 0.60–1.19 (moderate), 1.20–1.99 (large), and 2.00–3.99 (very large).

## Results

**Fig 2:**
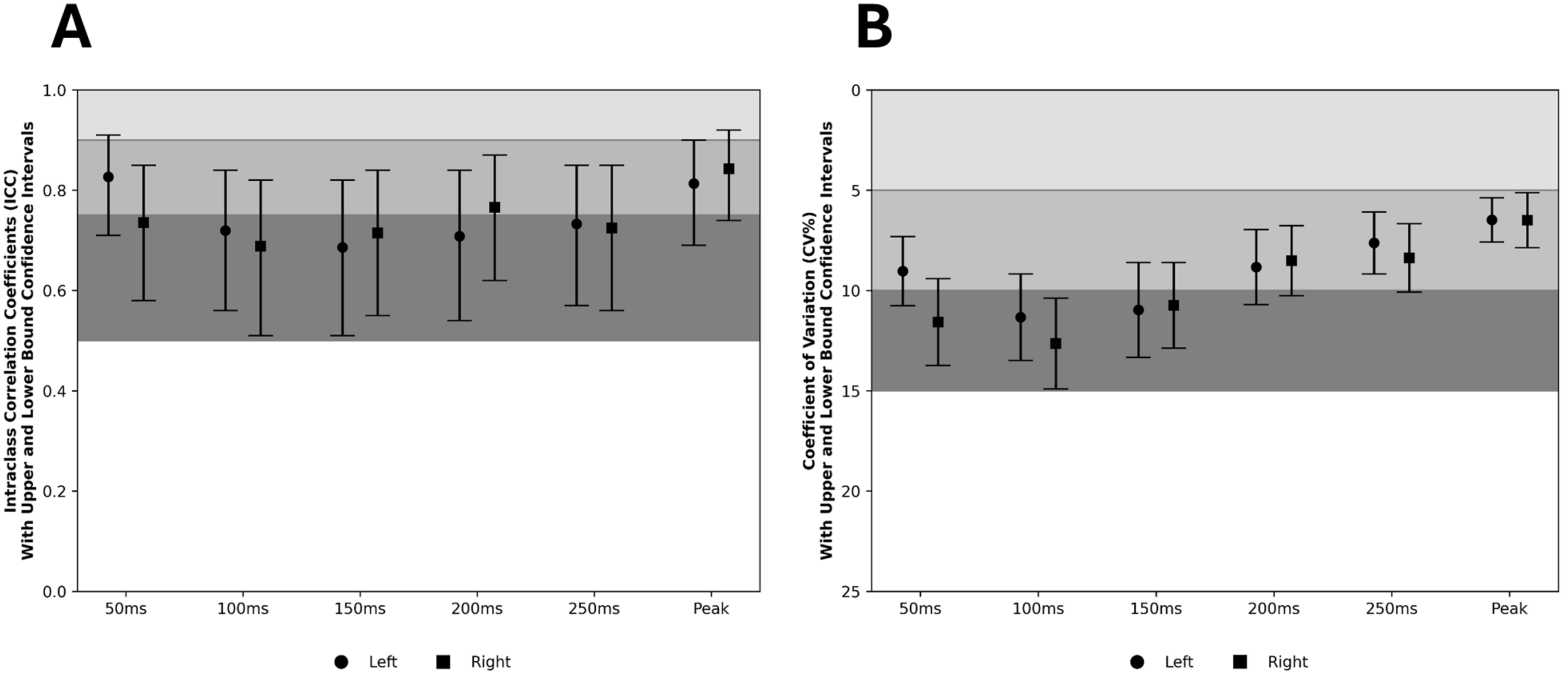
Visual representation of the between-session relative (ICC) and absolute reliability (CV%). Intraclass correlation coefficient (ICC) and coefficient of variation (CV) with 95% confidence intervals are presented for left (circles) and right (squares) limbs across six isometric hamstring force-time variables (50 ms, 100 ms, 150 ms, 200 ms, 250 ms, and Peak Force). Shaded regions represent ICC and CV reliability benchmarks (excellent-good-moderate).

Good-moderate relative and absolute reliabilities were observed across all early force-time variables (ICC > 0.51, CV% < 12.65%), with moderate relative and good absolute reliability for peak force assessments (ICC > 0.69, CV% < 6.50%) (Table 1; Fig 2a & 2b). No significant inter-session differences were observed for either limb between 50–200 ms. However, significant differences were found between sessions for right leg force at 250 ms (*p* < 0.05) and *p*eak force (*p* < 0.001). S*p*ecifically, the third session showed an increase in right leg force at 250 ms compared to the first session (*p* < 0.05; Hedges’ *g* = 0.35), and an increase on the third sessions com*p*ared to the second session (*p* < 0.001; *g* = 0.40) while no differences were observed for the left le*g*. At *p*eak force, si*g*nificant differences occurred between the first and third sessions (*p* < 0.05; *g* = 0.14) and between the second and third sessions (*p* < 0.001; *g* = 0.10), but not between the first and second sessions. Hed*g*es’ *g* effect sizes ranged from trivial-small across all force-time *p*oints (Table 1).

**Table 1:** Session mean force, standard deviation (SD), absolute (CV) and relative (ICC) reliability, and absolute measurement error (SEM; Newtons, MDD; Newtons) in 30 male collegiate athletes.

	Mean (SD)				Between Session Measures			
	Session 1	Session 2	Session 3	Hedges’ g Effect Size Ranges	ICC (95% CI)	CV (95% CI)	SEM (%)	MDD (%)
Peak Force (Left)	461.7 (81.0)	465.2 (72.4)	472.9 (79.6)	0.04;0.14	0.81 (0.69, 0.90)	6.48 (5.37, 7.58)	11.67 (2.5%)	32.35 (6.9%)
Peak Force (Right)	461.0 (79.4)	462.8 (81.2)	487.8 (68.7)	0.02;0.36	0.84 (0.74, 0.92)	6.50 (5.13, 7.87)	17.74 (3.8%)	49.17 (10.5%)
Force at 250 ms (Left)	390.2 (65.4)	377.9 (58.1)	389.6 (66.1)	0.01;0.20	0.73 (0.57, 0.85)	7.63 (6.08, 9.17)	11.62 (3.0%)	32.20 (8.3%)
Force at 250 ms (Right)	379.8 (69.9)	378.9 (60.1)	402.8 (57.7)	0.01;0.40	0.72 (0.56, 0.85)	8.38 (6.67, 10.08)	19.38 (5.0%)	53.72 (13.9%)
Force at 200 ms (Left)	380.8 (73.7)	362.1 (63.8)	367.3 (65.4)	0.08;0.27	0.71 (0.54, 0.84)	8.83 (6.97, 10.70)	13.31 (3.6%)	36.89 (10.0%)
Force at 200 ms (Right)	364.8 (76.2)	362.0 (62.7)	379.4 (64.5)	0.04;0.27	0.77 (0.62, 0.87)	8.51 (6.76, 10.26)	14.57 (4.0%)	40.39 (11.0%)
Force at 150 ms (Left)	326.1 (74.9)	318.7 (74.0)	319.6 (72.2)	0.01;0.10	0.69 (0.51, 0.82)	10.97 (8.60, 13.34)	21.18 (6.6%)	58.70 (18.3%)
Force at 150 ms (Right)	320.0 (75.5)	320.2 (68.4)	336.8 (60.2)	0.00;0.25	0.71 (0.55, 0.84)	10.73 (8.60, 12.87)	15.97 (4.9%)	44.27 (13.6%)
Force at 100ms (Left)	246.9 (59.3)	235.7 (60.0)	240.1 (52.8)	0.08;0.18	0.72 (0.56, 0.84)	11.33 (9.17, 13.49)	25.18 (10.5%)	69.79 (29.0%)
Force at 100ms (Right)	242.3 (61.6)	245.0 (59.2)	261.5 (56.9)	0.05;0.32	0.69 (0.51, 0.82)	12.65 (10.39, 14.91)	20.19 (8.1%)	55.98 (22.4%)
Force at 50 ms (Left)	182.9 (41.7)	176.4 (46.1)	181.4 (44.3)	0.03;0.15	0.83 (0.71, 0.91)	9.03 (7.30, 10.76)	10.42 (5.8%)	28.89 (16.0%)
Force at 50 ms (Right)	179.4 (44.2)	182.4 (48.7)	193.6 (42.5)	0.06;0.32	0.74 (0.58, 0.85)	11.57 (9.41, 13.74)	30.82 (16.6%)	85.43 (46.1%)

SD = standard deviation, ICC = intraclass correlation coefficient, CV = coefficient of variation, SEM = standard error of measurement, MDD = minimal detectable difference.

## Discussion

The primary aim of this study was to assess the between-session reliability of peak force and force at specific early time points in the isometric SL-LLB assessment which has yet to be established in the literature. The results indicate that force at early time points (<250 ms) can reliably be monitored longitudinally, demonstrating at least good to moderate reliability and only trivial to small effect sizes between sessions. Similarly, peak force exhibited good absolute and moderate relative reliability, reinforcing its consistency for repeated measures over time, similar to previously established assessments [9,49]. While significant differences were observed between sessions for the right limb at 250 ms and at peak force, the small effect sizes and the absence of a similar difference in the left limb suggests that the assessment remains reliable despite minor observed differences. Considering no observed differences were found between the first and second session, the isometric hamstring assessment may not require a familiarization session unlike other isometric assessments [50,51].

The findings of this study align with previously reported unilateral isometric hamstring assessments where reliability for peak force ranges from excellent-moderate [9,52–54] making it suitable for monitoring acute changes due to fatigue as well as chronic adaptations from training. Furthermore, unlike previously reported rapid force-generating capacities, the results of the present study demonstrate good to moderate between-session reliability for relative and absolute measures, which has yet to be reported in any isometric hamstring assessment emphasizing bi-articulate force production incorporating simultaneous emphasis on hip extension and knee flexion [12,29]. While early force production characteristics have been examined in knee-flexion-based hamstring assessments [9,49] similar reliability values ranging from good to moderate have been found. The present findings also reveal a gradual decline in reliability as the force assessment moves earlier in the force-time points, this trend likely occurs because rapid force production is more sensitive to fatigue-related fluctuations compared to peak force, which remains relatively stable across repeated assessments. Similar patterns are frequently observed in isometric assessments, whether single or multi-joint [9,55], where rapid force production is more susceptible to fatigue-induced declines. Rapid force production may also vary day-to-day depending on neuromuscular excitation or arousal levels, in addition to external factors such as fatigue or soreness which may further impact early force generating capacities. As a result, early force measures may exhibit lower reliability as could be seen in this study. Interestingly, force at 50 ms demonstrated higher reliability than most early force-time points, likely due to the influence of system mass on initial force output, with lower absolute forces at earlier time points being more heavily influenced by system mass

When comparing the results of the present study to that of more established multi-joint assessments like the isometric mid-thigh pull (IMTP), where peak force demonstrates good to excellent reliability [56] and early force-time points show poor to moderate reliability with similar protocols, but potentially improved reliability with shorter IMTP protocols [55] the isometric SL-LLB exhibits slightly worse performance at peak force although improved reliability at early time points when similar protocols are implemented. Overall, however, there is a high level of similarity. Considering the IMTP has been widely researched in sport performance monitoring [57] the findings of this study imply that the isometric SL-LLB could also serve as a potential reference point for assessing hamstring performance with the hope of mitigating injury risk. The slight advantage observed at early force-time points make it a valuable tool for capturing rapid force generation which as aforementioned, may be of pivotal importance especially for hamstring related injuries [58]. Although isometric hamstring assessments may have limited face validity due to the substantial evidence supporting eccentric muscle actions as the primary limiting factor in both performance [41] and injury mitigation [59], the advantages of isometric muscle assessments in mitigating DOMS and providing reliable rapid force generating capacities may outweigh these inherent limitations. Therefore, considering the importance for rapid force production to decelerate the tibia during sprinting [40], with knee extension angular velocities potentially exceeding 1200 deg/s [41] and ground contact times often under 100 ms [60], insufficient force production within these brief time frames may elevate injury risk and compromise performance. Therefore, the good-moderate reliability observed at early time points highlight the potential utility of the isometric SL-LLB for monitoring purposes. However, it may be best to capture force production at a minimum of 200 ms as anything lower only displays moderate reliability compared to good absolute reliability over 200 ms (CV% ≤ 8.83). This contrasts somewhat with prior within-session reliability research suggesting that force-time points above 150 ms may be sufficiently reliable [4], likely indicating that longitudinal assessments demands a more conservative threshold. Nonetheless, measuring isometric strength at force-time points as early as 200ms could offer valuable information into hamstring performance, with the hope of mitigating potential injury risk.

There are a few potential limitations to this study that should be considered. First, all testing was conducted during the athletes’ normal training week, which could result in residual fatigue even after the 48 hours of recovery between sessions. This residual fatigue may have influenced the athletes’ force production as their performance could be altered by ongoing training from other physical activities during the training week. This could potentially explain the reduced force production observed during the second testing day compared to the first, particularly across early force-time points. Second, the study was conducted during the first reporting week on campus for fall-based sports, as such, certain athletes may have experienced minor detraining due to a reduction in training load or cessation of training during their winter break. This detraining effect, which could reduce eccentric hamstring strength as early as two weeks after training cessation [61], could contribute to higher DOMS from a loss of the repeated bout effect, potentially leading to higher levels of fatigue and influenced force production during testing. This in turn, may explain higher DOMS levels during subsequent sessions within the week, potentially corroborated by the observed reduction in force on the second testing session in comparison with the first assessment. Moreover, the athletes in the study had little or no prior experience with force plate assessments, which may have impacted their understanding of the testing procedure, potentially leading to inconsistencies in performance due to unfamiliarity with the equipment and testing protocols. However, the trivial-small effect sizes observed between sessions suggests that any potential impact of familiarization would likely be minimal and not substantial enough to noticeably affect the results. Additionally, limb dominance was not recorded in the present study. Given the heterogenous sample spanning multiple sports, in which dominance patterns and their influence on force production may vary considerably, this represents a limitation when interpreting between-limb differences. Specifically, the observed asymmetries may reflect natural dominance-related variation rather than meaningful between-limb deficits, which could limit the application of these findings in injury risk screening contexts where asymmetry thresholds are used clinically. Future research would likely benefit from examining whether limb dominance influences between-session reliability within sport-specific populations, and whether dominant and non-dominant limbs demonstrate differential reliability profiles. Finally, the present study also included a male only sample, as sex-related differences in hamstring strength and reliability may exits, findings should be interpreted with caution when generalizing to female populations.

To the best of the authors’ knowledge, this is the first study to establish the between-session reliability values for the isometric SL-LLB while reporting early force-time characteristics of an isometric hamstring assessment that emphasizes simultaneous hip extension and knee flexion, as required during various athletic tasks. Following recent evidence demonstrating good within-session reliability of the isometric SL-LLB at later force-time points and highlighting the substantial influence of knee-joint angle on force-production outcomes [4], this study addresses the need to evaluate how the test can be applied and monitored longitudinally. Beyond longitudinal monitoring, the influence of individual characteristics such as height, body mass, limb length, and system mass warrants further examination to clarify how these factors impact force-production outcomes and improve interpretation of individual differences. Scaling approaches, including ratio scaling, allometric scaling, and expressing force relative to peak force, may help account for variation in body size and provide a clearer representation of rapid force production relative to an athlete’s strength capacities. Future research should investigate these factors in more detail to establish the most appropriate scaling methods and enhance the comparability of force-production measures across diverse athletic populations.

## Conclusions

The isometric SL-LLB bridge displays reliable between-session measurements across both peak force and early force-time points, supporting its use for longitudinal monitoring of hamstring strength. This assessment method may provide a practical tool for monitoring strength adaptations and may serve as a potential indicator of acute and chronic fatigue related decrements in performance. Regular monitoring of force production at various time points may help inform training decisions, recovery strategies, as well as return-to-play decision-making. Given the reliability and potential applicability beyond solely assessment-related implementations, and the broad implementation of isometric training for strength and neuromuscular adaptations, the SL-LLB may serve not only as a monitoring tool, but also a training intervention with inherent monitoring capabilities, making it a valuable addition to existing monitoring protocols in both training and rehabilitation environments.

## Supporting information

S1 Table Individual participant data by limb and session. (CSV)
